# Age modification of the relationship between C-reactive protein and fatigue: findings from *Understanding Society* (UKHLS)

**DOI:** 10.1017/S0033291717002872

**Published:** 2017-10-10

**Authors:** A. Hughes, M. Kumari

**Affiliations:** Institute for Social and Economic Research, University of Essex, Wivenhoe Park, Colchester, Essex, UK

**Keywords:** C-reactive protein, CRP, fatigue, idiopathic, inflammation, tiredness

## Abstract

**Background:**

Systemic inflammation may play a role in the development of idiopathic fatigue, that is, fatigue not explained by infections or diagnosed chronic illness, but this relationship has never been investigated in community studies including the entire adult age span. We examine the association of the inflammatory marker C-reactive protein (CRP) and fatigue assessed annually in a 3-year outcome period for UK adults aged 16–98.

**Methods:**

Multilevel models were used to track fatigue 7, 19, and 31 months after CRP measurement, in 10 606 UK individuals. Models accounted for baseline fatigue, demographics, health conditions diagnosed at baseline and during follow-up, adiposity, and psychological distress. Sensitivity analyses considered factors including smoking, sub-clinical disease (blood pressure, anaemia, glycated haemoglobin), medications, ethnicity, and alcohol consumption.

**Results:**

Fatigue and CRP increased with age, and women had higher values than men. CRP was associated with future self-reported fatigue, but only for the oldest participants. Thus, in those aged 61–98 years, high CRP (>3 mg/L) independently predicted greater fatigue 7, 19, and 31 months after CRP measurement [odds ratio for new-onset fatigue after 7 months: 1.88, 95% confidence interval (CI) 1.21–2.92; 19 months: 2.25, CI 1.46–3.49; 31 months: 1.65, CI 1.07–2.54]. No significant longitudinal associations were seen for younger participants.

**Conclusions:**

Our findings support previously described CRP–fatigue associations in older individuals. However, there are clear age modifications in these associations, which may reflect a contribution of unmeasured sub-clinical disease of limited relevance to younger individuals. Further work is necessary to clarify intervening processes linking CRP and fatigue in older individuals.

## Introduction

### Idiopathic fatigue and possible causes

In healthy individuals, fatigue is usually experienced as a transient response to physical exertion or prolonged activity, which reduces with rest and does not normally interfere with daily tasks (Kluger *et al.* 2013). However, it can also be persistent, and not clearly attributable to exertion: this kind of ongoing fatigue can disrupt quality of life, as well as social or occupational functioning (Finsterer & Mahjoub, 2014). It is a substantial driver of contact with primary care services, estimated as the primary reason for around 6.5% of general practitioner visits, and a secondary reason for a further 19% of visits (Cullen *et al.* 2002). While fatigue is more commonly reported by women than men (Newton & Jones, 2010; Blackwell & Clarke, 2013), its relationship with age is unclear; some studies report age-related increases in reported fatigue, but others report age-related decreases (Blackwell & Clarke, 2013; Dolan & Kudrna, 2015). Fatigue is sometimes a consequence of chronic illnesses; it is a common symptom of conditions including cancer (Bower, 2014), Parkinson's disease (Friedman *et al.* 2007), and diabetes (Lasselin *et al.* 2012). However, in studies of primary care patients presenting with fatigue, diagnoses of relevant underlying illness are subsequently made only in a minority of cases (Nijrolder *et al.* 2009) and only slightly more than for controls (Stadje *et al.* 2016). Hence, many cases of fatigue have no obvious explanation, and as such are termed ‘idiopathic’ (Alexander *et al.* 2010). It has been suggested that low-grade inflammatory activity may play a role in otherwise unexplained fatigue (Cho *et al.* 2009; Alexander *et al.* 2010; Cho *et al.* 2013), based on experiments showing that exogenous inflammatory agents can induce fatigue in healthy humans (Spath-Schwalbe *et al.* 1998), and the fact that fatigue is a key component of the ‘sickness behaviour’ triggered by the acute inflammatory response to infection or injury (Dantzer *et al.* 2008; Miller *et al.* 2009). From this, it has been proposed that ‘systemic’, ‘chronic’ or ‘low-grade’ inflammation – a milder, temporally extended analogue of the acute inflammatory response to infection or injury that occurs in the absence of these triggers (Pearson *et al.* 2003) – may be involved in the development of idiopathic fatigue. Typically higher in women, systemic inflammation increases with age (Krabbe *et al.* 2004). Though associated with many chronic illnesses (Gan *et al.* 2004; Dowlati *et al.* 2010), systemic inflammation can also occur in the absence of disease; it is strongly and positively influenced by smoking and adiposity (Hamer *et al.* 2009; Howren *et al.* 2009), with associations also shown for stressful events and experiences (Yudkin *et al.* 2000; Steptoe *et al.* 2007).

### Previous studies of inflammation and idiopathic fatigue

Most existing studies on inflammatory markers and fatigue are of limited relevance to idiopathic fatigue, describing associations with fatigue severity in the context of cancer, multiple sclerosis or other specific illnesses using small clinical samples (Bower, 2014; Patejdl *et al.* 2016). In community samples, inflammation–fatigue associations may still be confounded by chronic illness; a reverse-causal pathway from fatigue to inflammation may also operate via reductions in physical activity, which has anti-inflammatory effects (Valentine *et al.* 2011). Longitudinal analysis can help clarify these issues by modelling later fatigue as a function of earlier inflammation, with adjustment for chronic illness. Since fatigue-like symptoms form part of depressive symptomatology, with which a more general association of systemic inflammation is established (Dowlati *et al.* 2010), associations must also be shown to be independent of depressive symptoms. Lastly, since both fatigue and inflammatory activity are elevated following injury or infection (Vollmer-Conna *et al.* 2004), steps must be taken to isolate systemic inflammation from transient processes. Typically, researchers exclude participants with C-reactive protein (CRP) >10 mg/L (Pearson *et al.* 2003), although this cut-off may be conservative (Ishii *et al.* 2012).

To our knowledge, only two longitudinal studies have examined inflammation–fatigue associations in large community samples. The first, based on 2983 US adults aged 33–45, reported that the inflammatory marker CRP was associated with fatigue 5 years later and *vice versa*, which the authors interpret as supporting a bidirectional relationship (Cho *et al.* 2009). The second, in 4847 British Civil servants aged 39–63, found onset of fatigue during a 3-year follow-up was predicted by higher levels of two inflammatory markers (CRP and interleukin-6) at baseline (Cho *et al.* 2013). While these studies are consistent with a role for systemic inflammation in idiopathic fatigue, they had several shortcomings. Firstly, since neither study excluded participants with very high CRP values, associations may have been inflated by infection- or injury-related fatigue, rather than the chronic processes ostensibly under investigation. Secondly, both samples were considerably age-restricted, meaning this relationship is yet to be examined in individuals younger than 33 or older than 63. Thirdly, since both studies consider only a single follow-up point, the temporal evolution of fatigue following systemic inflammation is unknown. Given the high possibility of confounding and reverse causation in this relationship, mapping such ‘trajectories’ across multiple follow-up points could be illuminating, since a pattern of decreasing associations may indicate reverse causation or confounding by undiagnosed conditions at baseline. As the 2013 study adjusted for chronic illness diagnosed before baseline but not during follow-up, previously reported inflammation–fatigue associations in middle age may have been inflated by chronic disease not yet diagnosed at baseline. We aim to fill these gaps using data from a large, nationally representative survey of UK adults aged 16–98. Using three follow-up points, we map trajectories of fatigue 1, 2, and 3 annual waves after CRP measurement; exclude individuals with signs of acute inflammation; consider newly diagnosed conditions at each follow-up point; and explore differential effects by age and gender.

## Methods

### Participants

The UKHLS is an annual longitudinal survey of over 40 000 UK households, beginning in 2009–2010. It comprises a general population sample (GPS), a stratified clustered random sample of households representative of the UK population, and a smaller component from the pre-existing British Household Panel Survey (BHPS) (Knies, 2015). Annual interviews collecting sociodemographic information are conducted throughout each year, usually in the same month for each participant. Biomedical measures and blood samples were collected during a single nurse visit, which took place in the participant's home 5 months after the annual wave 2 interview (GPS participants, 2010–2012) or wave 3 interview (BHPS participants, 2011–2013) (McFall *et al.* 2014).

Ethical approval for the Understanding Society nurse visit was obtained from the National Research Ethics Service (Reference: 10/H0604/2). Participants gave written consent for blood sampling (McFall *et al.* 2014). Respondents were eligible for a nurse visit if they had taken part in that wave's main interview in English; were aged 16+; lived in England, Wales or Scotland; and were not pregnant. Of 35 875 participants meeting these requirements, 57.5% took part. The initial sample for this analysis was defined as participants present at the biomedical assessment and at least one subsequent wave. Of 19 769 participants in the initial sample, 12 429 (62.9%) had usable CRP measurements. Missingness was usually due to non-consent for blood samples, more rarely because participants were taking anti-coagulant drugs, had a bleeding disorder or other reasons; details are provided in the user guide (Benzeval *et al.* 2014). Three hundred and sixty lacked a usable fatigue measurement at follow-up, and 870 were excluded for missing covariates. Excluding 593 participants with CRP >10 mg/L, including 399 between 10 and 20 mg/L, left a final sample of 10 606 participants and 28 407 observations.

### Measures

#### CRP

CRP was analysed from serum by Newcastle upon Tyne Hospitals NHS Foundations Trust, using the high-sensitivity N Latex CRP mono Immunoassay on the Behring Nephelometer II Analyser. Intra-assay coefficients of variation were <2% (Benzeval *et al.* 2014). For analysis, CRP was categorised as low (<1.00 mg/L), medium (1.00–2.99 mg/L), and high (3.0–10.0 mg/L), in accordance with the Centres for Disease Control and Prevention/American Heart Association recommendations (Pearson *et al.* 2003) and previous analysis on this topic (Cho *et al.* 2009).

#### Fatigue

At each wave, participants completed the 12-item version of the Short Form Health Survey (SF-12), designed to capture overall health, pain, fatigue, and health-related limitations to functioning in persons over 14 (Ware & Sherbourne, 1992). In the 12-item version, fatigue is indexed by a single item: ‘*How much of the time during the past 4 weeks did you have a lot of energy*?’ This is one of four vitality sub-scale questions from the 36-item version of the Short Form Health Survey (SF-36), of which the 12-item version has been shown to be a reasonable substitute (Jenkinson *et al.* 1997), and the same question used in a previous longitudinal analysis on this topic (Cho *et al.* 2009). Participants could respond using options listed on a showcard: all of the time; most of the time; some of the time; a little of the time; none of the time. As responses were ordered and their distribution approximated a normal curve, fatigue was considered a quasi-interval variable and treated as continuous in linear regressions, consistent with the procedure of the earlier study (Cho *et al.* 2009). For each time point, a binary fatigue measure was formed by combining participants who reported having a lot of energy ‘a little of the time’ and ‘none of the time’ into a high-fatigue group, and participants who reported having a lot of energy ‘all’, ‘most’, or ‘some’ of the time into a low-fatigue group. This allowed analysis of onset of binary fatigue during follow-up, an approach taken by the other existing paper on this topic (Cho *et al.* 2013). For descriptive purposes, ‘low’-, ‘mid’-, and ‘high’-fatigue groups were defined as participants reporting having a lot of energy ‘all’ or ‘most’ of the time; ‘some’ of the time; or ‘a little’ or ‘none’ of the time.

#### Covariates

All models took account of baseline fatigue from the annual interview 5 months before CRP measurement. Hence, linear models adjusted for baseline fatigue, while in logistic models of binary fatigue onset, analysis was restricted to people without binary fatigue at baseline. Age and gender were reported by questionnaire at the biomedical assessment, with age tertiles calculated as 16–43, 44–60, and 61–98; within each tertile age in years was adjusted for. Equivalised household income was derived from questionnaire information from the annual interview corresponding to the biomedical assessment, from which age-band-specific quartiles were calculated. Recent diagnoses at baseline of relevant somatic illness were derived using questionnaire information from UKHLS W1 and W2 (GPS participants) or BHPS W18, UKHLS W1, and UKHLS W2 (BHPS participants). This included asthma, emphysema, chronic bronchitis, cancer, cardiac problems, high blood pressure, and diabetes. The role of new somatic illness was explored using diagnoses of these conditions plus arthritis at each follow-up wave. Arthritis at baseline could not be included, as the BHPS component of the sample was not asked about arthritis until UKHLS W2. All models adjusted for psychological distress at outcome, using a summary score (possible range 0–36) from the 12-item General Health Questionnaire (GHQ). Baseline body mass index (BMI) was calculated from height and weight measured by the nurse at the biomedical visit, using a portable stadiometer, and the Tanita BF522 digital floor scale, respectively (McFall *et al.* 2014). BMI was classified using WHO categories: recommended weight (18.5–24.9), overweight (25.0–29.9), obese (⩾30) or underweight (<18.5). At the nurse assessment, participants were asked to name up to 22 currently prescribed medicines, and containers checked by the nurse. Medications potentially affecting CRP included non-steroidal anti-inflammatory medications (NSAIDs), statins, corticosteroids, oral contraceptives, and hormone replacement therapy (HRT). Self-reported ethnicity was categorised as white (British, Irish or other white) and any other ethnicity, including mixed (3.8% of the final sample). Glycated haemoglobin (HbA1c) was defined as normal (<42 mmol/mol), prediabetes (42–47 mmol/mol), and diabetes (⩾48 mmol/mol). Hypertension (⩾140 systolic, ⩾90 diastolic) was calculated from blood pressure measured by the nurse, using the Omron HEM 907 machine. Anaemia, from blood samples, was defined as a haemoglobin value <12 g/dL for women and <13 g/dL for men. Information on smoking and alcohol consumption came from wave 2 questionnaires for all participants. We used both drinking frequency (days in the past week when alcohol was consumed, categorised as none, 1–2, 3–4, or 5+) and the maximum alcoholic units consumed on any day of the past 7 (none/⩽4.0/4.1–6.0/6.1–8.0/8.1–10.0/10.1–15.0/>15.0).

### Analysis

In adjusted cross-sectional models, significant interaction terms between CRP categories (low/medium/high) and both age in years (*p* = 0.001, *p* < 0.001 for medium*age and high*age, respectively) and age tertiles (*p* = 0.06, *p* = 0.02, *p* = 0.06, *p* = 0.009 for medium*44–60, medium*61–98, high*44–60, and high*61–98, respectively) pointed to age modification, but there was no evidence of gender interaction (*p* = 0.39 for medium*female and 0.76 for high*female). Longitudinal models were therefore calculated separately by age tertiles, but not gender.

To map trajectories of CRP–fatigue associations, fatigue measurements 1, 2, and 3 annual waves post-baseline were modelled as functions of CRP 5 months after baseline fatigue, such that outcome points were 7, 19, and 31 months after CRP measurement. Data were reshaped to make each outcome fatigue measurement a separate observation, and a repeated-measures, multilevel panel data structure specified, with fatigue measurements 7, 19, and 31 months after CRP measurement nested within a level representing individuals. To separately investigate trajectories by age tertile, a three-way interaction term was used, linking age tertiles (16–43, 44–60, and 61–98), CRP groups (<1.00, 1.00–2.99, 3.00–10), and waves since baseline. Models treating fatigue continuously used STATA's xtreg command; models investigating odds of binary fatigue onset used STATA's xtlogit command, and were restricted to individuals without binary fatigue at baseline (*N* = 8531). Age in years, gender, household income, BMI, baseline fatigue, and somatic illnesses at baseline were included as time-invariant covariates, GHQ at outcome and new diagnoses during follow-up as time-varying covariates. Unbalanced data were allowed, meaning participants were included even if not present at all outcome waves. All analyses used robust standard errors to account for survey effects.

#### Sensitivity analyses

Since the threshold of CRP 10 mg/L for separating chronic from infection-related inflammation may be conservative (Ishii *et al.* 2012), we repeated analyses including 399 individuals with CRP between 10 and 20 mg/L. Additional adjustment explored the impact of time of day, season, and processing time of blood samples, plus several factors with substantial additional missingness: HbA1c, hypertension, anaemia status, and alcohol consumption measures. Further sensitivity analyses examined the impact of anti-inflammatory medications, oral contraceptives, and HRT by excluding participants taking these medications. Only 3.8% of participants defined as non-white or mixed ethnicity, precluding adjustment for ethnicity, so its impact was explored by excluding the small group of non-white or mixed participants. Smoking influences CRP, and may therefore indirectly affect fatigue via CRP. For this reason, smoking was not included as a covariate in the main models to avoid overadjustment (i.e. adjustment for a variable upstream of the exposure on the causal pathway), but its impact was explored in two sensitivity analyses. In the first, we adjusted for five-category smoking status (never, ex-, and banded current), and in the second current smokers were excluded entirely.

## Results

### Descriptive

Participants excluded for missing data, compared to those retained, did not differ significantly by gender or BMI. However, they were older (41.0% *v.* 33.4% aged 61–98, mean age 54.8 *v*. 51.6), with slightly higher CRP (2.13 *v.* 2.01 mg/L), and slightly higher fatigue at all points (e.g. 2.86 *v.* 2.66 at baseline). They were more likely to have a long-term illness at baseline (35.6% *v.* 30.8%) and to be taking CRP-relevant medication (26.9% *v.* 20.3%). Participants excluded for CRP >10 mg/L were more often female (63.9% *v.* 55.3%), older (40.6% *v.* 33.4% aged 61–98, mean age 54.6 *v.* 51.6), heavier (BMI 31.4 *v.* 27.8), with greater fatigue at all points (e.g. 3.08 *v.* 2.66 at baseline). They were more likely to have a long-term illness at baseline (45.7% *v.* 30.8%), to be taking CRP-relevant medications (32.0% *v.* 20.3%), and to smoke (25.8% *v.* 18.3%). Their CRP ranged from 10.1 to 228 mg/L, with a mean of 22.7 mg/L.

In the final sample, CRP increased with age, and in every age group was higher for women than men; mean CRP values for men aged 16–43, 44–60, and 61–98 were 1.45, 1.90, and 2.16 mg/L, and for women aged 16–43, 44–60, and 61–98, they were 1.91, 2.08, and 2.44 mg/L. However, within CRP categories, mean CRP was comparable across age tertiles: mean low, mid, and high CRP values for the 16–43 group were 0.48, 1.71, and 5.16; for the 44–60 group, they were 0.52, 1.74, and 5.18; and for the 61–98 group, they were 0.57, 1.76, and 5.18.

Fatigue increased with age, and within each age group women reported more fatigue than men. At baseline, the proportion of participants reporting binary fatigue was: 11.5% (men aged 16–43 years), 13.3% (women 16–43), 16.6% (men 44–60), 16.9% (women 44–60), 19.5% (men 61–98), and 21.7% (women 61–98). Table 1 shows sample descriptive characteristics by fatigue level. Participants experiencing greater fatigue were more likely to have diagnosed illnesses at baseline, take anti-inflammatory medications, smoke regularly and more heavily, have extreme BMIs including underweight, and have HbA1c levels indicating prediabetes or diabetes (*p* values from χ^2^ for trend all <0.05). Fatigue did not differ significantly by ethnicity, blood pressure or use of oral contraceptives or HRT.

**Table 1.** Descriptive characteristics of analytic sample (N = 10 606) by fatigue at baseline

	Low fatigue^a^	Mid fatigue^a^	High fatigue^a^
Baseline characteristics	Mean (s.d.)	Mean (s.d.)	Mean (s.d.)
Age (years)	50.6 (16.3)	51.7 (17.1)	54.3 (17.4)
GHQ score (possible range 0–36)	8.9 (3.6)	11.5 (4.7)	16.0 (7.0)
C-reactive protein	(%)	(%)	(%)
Low (<1 mg/L)	44.8	37.2	31.3
Mid (1–2.99 mg/L)	37.9	38.5	37.3
High (3.0–10.0 mg/L)	17.3	24.3	31.5
Gender			
Male	48.8	42.7	36.8
Female	51.2	57.3	63.3
Ethnicity			
White (British/Irish/other white)	95.4	95.6	96.5
Any other ethnicity	3.8	3.9	3.2
Missing	0.8	0.5	0.3
Somatic diagnoses (respiratory disease, cancer, cardiac disease, high blood pressure, diabetes)			
No	76.6	66.1	53.3
Yes	23.4	33.9	46.7
Body mass index			
<18.5	2.3	3.7	5.6
18.5–24.9	32.8	27.0	22.8
25.0–29.9	41.8	38.2	32.5
30–34.9	17.0	21.3	22.8
>35	6.1	9.9	16.2
Smoking status			
Never	55.2	52.0	45.5
Ex	27.2	27.6	29.8
Current, ⩽10/day	8.4	9.6	9.9
Current, 11–20/day	6.2	7.9	10.2
Current, >20/day	1.1	1.3	2.7
Missing	1.9	1.6	1.9
Takes NSAIDs, corticosteroids or statins			
No	84.9	78.5	66.9
Yes	15.1	21.5	33.1
Takes HRT or oral contraceptives			
No	94.0	94.5	94.4
Yes	6.0	5.5	5.6
Anaemia (haemoglobin <12 g/L for women, <13 g/L for men)			
No	85.1	83.8	79.5
Yes	8.4	9.5	13.1
Missing	6.5	6.7	7.5
Hypertension (⩾140 systolic, ⩾90 diastolic)			
Normotensive untreated	58.5	52.6	43.4
Normotensive treated	8.6	13.2	17.2
Hypertensive	4.1	4.2	4.3
Missing	28.9	30.0	35.1
Glycated haemoglobin			
Normal (<42 mmol/mol)	85.1	81.3	73.8
Prediabetes (42–47 mmol/mol)	5.4	7.3	9.5
Diabetes (⩾48 mmol/mol)	3.1	5.2	9.3
Missing	6.4	6.3	7.5

GHQ, General Health Questionnaire.

a Fatigue groups categorised as: low fatigue: a lot of energy ‘all’ or ‘most’ of the time; mid fatigue: a lot of energy ‘some’ of the time; high fatigue: a lot of energy ‘a little’ or ‘none’ of the time.

### Cross-sectional models

For participants aged 16–43, neither medium nor high CRP was associated with greater fatigue [coeff: −0.02, confidence interval (CI) 0.08–0.22, *p* = 0.46 and coeff: 0.06, −0.2 to 0.13, *p* = 0.12, respectively]. For participants aged 44–60, high CRP was associated with greater fatigue (coeff: 0.15, CI 0.08–0.22); for participants aged 61–98, medium and high CRP were associated with greater fatigue (coeff: 0.08, CI 0.02–0.14, coeff: 0.19, 0.12–0.27). Only when participants with CRP between 10 and 20 mg/L were included, significant cross-sectional associations were seen with high CRP in every age group (coeff: 0.07, CI −0.00 to 0.14, *p* = 0.05), 44–60 (coeff: 0.17, CI 0.10–0.24, *p* < 0.001), and 61–98 (coeff: 0.22, CI 0.15–0.29, *p* < 0.001) (Table 2).

**Table 2.** Fully adjusted cross-sectional associations of C-reactive protein (CRP) and fatigue^a^

CRP category	Coeff	CI	*p*
Age 16–43			
Mid (1.00–2.99 mg/L)	−0.02	−0.08–0.03	0.46
High (3.00–10.0 mg/L)	0.06	−0.02–0.13	0.12
Age 44–60			
Mid (1.00–2.99 mg/L)	0.05	−0.01–0.11	0.06
High (3.00–10.0 mg/L)	0.15	0.08–0.22	<0.001
Age 61–98			
Mid (1.00–2.99 mg/L)	0.08	0.02–0.14	0.01
High (3.00–10.0 mg/L)	0.19	0.12–0.27	<0.001

a Adjusted for age in years, gender, long-term illness, psychological distress, BMI and household income.

Low CRP (<1.00 mg/L) is the reference in all models.

### Longitudinal models

In adjusted multilevel models, significant CRP-associated elevations were seen only for participants aged 61–98 (Table 3), for whom high CRP (>3 mg/L) was significantly associated with elevated fatigue at all follow-up points, and mid-CRP (1.00–3.00 mg/L) associated with elevated fatigue at the second and third outcome points. While CIs overlapped, the association with high CRP decreased across follow-up, from 0.16 (CI 0.10–0.23) one wave post-baseline to 0.08 (CI 0.01–0.16) three waves post-baseline, but the association with mid-CRP did not [0.02 (CI −0.03 to 0.08) one wave post-baseline, 0.07 (CI 0.01–0.13) two waves post-baseline, and 0.09 (CI 0.00–0.18) three waves post-baseline]. Similarly, significant CRP-associated elevation in odds of fatigue onset was seen only for participants aged 61–98, and only with high CRP. The association strengthened at the second outcome point, from odds ratio (OR) 1.88 (CI 1.21–2.92) to OR 2.25 (CI 1.46–3.49) before falling to OR 1.65 (CI 1.07–2.54).

**Table 3.** Multilevel models

Fully adjusted associations of CRP and fatigue: 1, 2, and 3 waves post-baseline^a^ (*N* = 10 606)									
Tertile 1: 16–43 *N* = 3473	1 wave later (7 months after CRP measurement)			2 waves later (19 months after CRP measurement)			3 waves later (31 months after CRP measurement)		
CRP category	Coeff	CI	*p*	Coeff	CI	*p*	Coeff	CI	*p*
Mid (1.00–2.99 mg/L)	0.00	−0.06 to 0.05	0.91	0.00	−0.05 to 0.06	0.86	0.03	−0.03 to 0.09	0.34
High (3.00–10.0 mg/L)	0.05	−0.02 to 0.12	0.14	−0.00	−0.07 to 0.07	0.99	0.04	−0.03 to 0.11	0.27
Tertile 2: 44–60 *N* = 3588	1 wave later (7 months after CRP measurement)			2 waves later (19 months after CRP measurement)			3 waves later (31 months after CRP measurement)		
CRP category	Coeff	CI	*p*	Coeff	CI	*p*	Coeff	CI	*p*
Mid (1.00–2.99 mg/L)	0.01	−0.04 to 0.06	0.65	0.03	−0.02 to 0.08	0.29	0.05	−0.03 to 0.14	0.23
High (3.00–10.0 mg/L)	0.03	−0.03 to 0.10	0.30	0.05	−0.02 to 0.12	0.16	0.02	−0.04 to 0.09	0.46
Tertile 3: 61–98 *N* = 3545	1 wave later (7 months after CRP measurement)			2 waves later (19 months after CRP measurement)			3 waves later (31 months after CRP measurement)		
CRP category	Coeff	CI	*p*	Coeff	CI	*p*	Coeff	CI	*p*
Mid (1.00–2.99 mg/L)	0.02	−0.03 to 0.08	0.45	0.07	0.01 to 0.13	0.03	0.09	0.00 to 0.18	<0.05
High (3.00–10.0 mg/L)	0.16	0.10 to 0.23	<0.001	0.14	0.06 to 0.21	<0.001	0.08	0.01 to 0.16	0.03
Fully adjusted associations of CRP and onset of fatigue: 1, 2, and 3 waves later^b^ (*N* = 8813)									
Tertile 1: 16–43 *N* = 2968	1 wave later (7 months after CRP measurement)			2 waves later (19 months after CRP measurement)			3 waves later (31 months after CRP measurement)		
CRP category	OR	CI	*p*	OR	CI	*p*	OR	CI	*p*
Mid (1.00–2.99 mg/L)	0.77	0.53–1.11	0.17	0.86	0.56–1.27	0.45	1.17	0.79–1.72	0.43
High (3.00–10.0 mg/L)	0.73	0.45–1.17	0.19	0.89	0.56–1.41	0.62	1.39	0.89–2.17	0.15
Tertile 2: 44–60 *N* = 2986	1 wave later (7 months after CRP measurement)			2 waves later (19 months after CRP measurement)			3 waves later (31 months after CRP measurement)		
CRP category	OR	CI	*p*	OR	CI	*p*	OR	CI	*p*
Mid (1.00–2.99 mg/L)	1.34	0.89–2.02	0.16	1.05	0.72–1.54	0.80	1.43	0.76–2.67	0.27
High (3.00–10.0 mg/L)	1.35	0.82–2.20	0.22	1.43	0.92–2.21	0.11	1.27	0.80–2.02	0.31
Tertile 3: 61–98 *N* = 2859	1 wave later (7 months after CRP measurement)			2 waves later (19 months after CRP measurement)			3 waves later (31 months after CRP measurement)		
CRP category	OR	CI	*p*	OR	CI	*p*	OR	CI	*p*
Mid (1.00–2.99 mg/L)	1.19	0.79–1.78	0.41	1.31	0.88–1.95	0.18	1.78	0.94–3.37	0.08
High (3.00–10.0 mg/L)	1.88	1.21–2.92	0.005	2.25	1.46–3.49	<0.001	1.65	1.07–2.54	0.02

a All models adjust for fatigue at baseline, age in years, gender, household income, BMI, and long-term illness at baseline, new somatic illness, and psychological distress at follow-up. Low CRP (<1.00 mg/L) is the reference group in all models.

b All models adjust for age in years, gender, household income, BMI, and long-term illness at baseline, new somatic illness, and psychological distress at follow-up. Low CRP (<1.00 mg/L) is the reference group in all models.

### Sensitivity analyses

Including participants with CRP 10–20 mg/L, in the 44–60 group, high CRP predicted higher fatigue only at the second outcome point in linear (coeff 0.07, CI 0.00–0.13, *p* = 0.04) and logistic models (OR 1.56, CI 1.02–2.36, *p* = 0.04), with no associations seen in the 16–43 group. Only by imposing no upper limit for CRP, and with diagnoses during follow-up ignored, did high CRP in the 44–60 group predict 1, 2, and 3 waves later, both elevated fatigue (coeff 0.06, CI 0.00–0.12, *p* = 0.05; coeff: 0.09, CI 0.02–0.15, *p* = 0.009; coeff: 0.07, 0.01–0.15, *p* = 0.03), and new-onset fatigue (OR 1.52, CI 0.97–2.39, *p* = 0.07; OR 1.72, CI 1.14–2.58, *p* = 0.009; OR 1.70, CI 1.10–2.61, *p* = 0.01). Minimal differences were made by adjustment for five-group smoking status, time of day, season and processing time of blood samples, and for anaemia status, HbA1c, nurse-measured blood pressure, and both measures of alcohol intake (online Supplementary Tables S1 and S2). Conclusions were unaffected by exclusion of non-white or mixed participants, participants taking medications, and current smokers.

## Discussion

We find the association of fatigue and CRP levels indicative of systemic inflammation to be substantially modified by age. Thus in cross-sectional models, both medium and high CRPs are associated with fatigue in participants aged 61–98, only high CRP is associated with fatigue in participants aged 44–60, and neither is associated with fatigue in participants aged 16–43, despite comparable within-group CRP values across age tertiles. Longitudinally, both medium and high CRPs predict elevated fatigue, and high CRP predicts odds of new-onset fatigue, in participants aged 61–98 following adjustment for a wide variety of covariates. No such associations are seen in younger groups.

### Cross-sectional associations

The cross-sectional associations observed in the middle and oldest age groups are consistent with previous studies reporting cross-sectional CRP–fatigue associations (Cho *et al.* 2009; Cho *et al.* 2013). However, an association in younger participants emerged only with inclusion of participants with CRP of 10–20 mg/L. This suggests that the association reported in a previous study may at least partly reflect transient inflammation due to current infections or injury. Alternatively, a threshold effect may exist, with chronically elevated CRP in younger individuals associated with fatigue only at very high values. Discrepant results may also relate to differing characteristics of participants in the UK and America, for instance different BMI profiles for this age group. In the previous study (Cho *et al.* 2009), mean BMI for participants reporting low, intermediate, and high fatigue were 27.6, 29.1, and 30.7 kg/m^2^, respectively. In our sample, mean BMI in equivalent groups aged 33–45 were lower at 27.3, 28.6, and 28.4 kg/m^2^.

### Longitudinal associations

In men and women aged 61–98, high CRP (3–10 mg/L) predicted higher fatigue and increased odds of binary fatigue onset 7, 19, and 31 months later. Results in this group are therefore consistent with previous longitudinal studies reporting robust predictive associations at single outcome points 3 or 5 years later (Cho *et al.* 2009, 2013), and indicate that predictive associations are visible earlier than previously shown. Building on those studies, we do not see declining CRP–fatigue associations after the first outcome measurement at 7 months, a pattern which would suggest reverse causation, that is, influence of fatigue on CRP. CRP–fatigue associations in the 61–98 group were robust to adjustment for GHQ, and hence do not appear to simply reflect a broader connection between inflammation and psychological distress. We were able to show that CRP–fatigue associations in this group are independent of chronic disease diagnoses during follow-up as well as at baseline, with sensitivity analyses indicating they are independent of smoking, and various aspects of sub-clinical health. Since participants excluded due to missing data were older and in poorer health than those retained, with higher CRP and greater fatigue, any bias from their exclusion is likely to have underestimated associations in that group, such that estimates are conservative.

### Age modification

An unexpected result was the lack of longitudinal CRP–fatigue associations for participants aged 60 or younger. This contrasts with previous studies using similar fatigue measures, reporting associations in participants aged 33–45 (Cho *et al.* 2009) and 39–63 (Cho *et al.* 2013). A crucial difference is that unlike those studies, we excluded participants with CRP values indicative of acute inflammation in order to focus on chronic processes. We also adjusted for chronic illness diagnosed during follow-up. Including CRP between 10 and 20 mg/L, high CRP predicted fatigue only at the second outcome point for those aged 44–60. Only with no upper limit for CRP, and diagnoses during follow-up ignored, did high CRP in the 44–60 group predict elevated fatigue at all outcome points. Our results therefore suggest previously reported longitudinal CRP–fatigue associations in middle age, interpreted as evidence for a role of systemic inflammation itself in fatigue development, may have been inflated by infection-related sickness behaviour and/or chronic illness soon to be diagnosed.

Why would CRP linked to chronic processes be clearly associated with future fatigue only in older individuals? Like in the previous studies, CRP was calculated from a single measurement. This may have biased downwards CRP–fatigue associations, since a single measurement of an inflammatory marker leads to more ‘noise’ than when two measurements are averaged (Kivimaki *et al.* 2014). However, it is unclear why such measurement error would affect estimates only in younger groups. Since our fatigue measure was a subjective, self-reported measure, it is possible that younger and older participants interpreted or responded to the question in systematically different ways, a phenomenon observed for more global assessments of health (Shooshtari *et al.* 2007). This could produce different observed relationships, even if the biological processes involved do not differ by age. Since this study's outcome was self-reported fatigue and not fatigability, it is also possible that age-group differences in physical activity – which is tiring, but also has anti-inflammatory effects – may have modified inflammation–fatigue associations. Since objective measures of physical activity (e.g. from pedometer readings) were not available in this dataset, this hypothesis cannot be tested further in this study population. A final possibility is that inflammation–fatigue associations in older participants are explained by aspects of sub-clinical disease by which younger people are only rarely affected. Although we examined several potential biological mediating pathways – blood pressure, HbAa1c, haemoglobin levels – and found they did not explain associations, other plausible pathways could not be examined as they were not measured in the study. A plausible candidate is atherosclerosis, given that inflammation is involved in the formation of arterial plaques (Libby, 2012); it is for this reason, it is believed, that inflammatory markers independently predict cardiac events among apparently healthy people (Bassuk *et al.* 2004). If idiopathic fatigue in older people is explained by atherosclerosis, of which CRP is an established marker (Kivimäki *et al.* 2008), we speculate that fatigue may be an underappreciated and easily obtainable measure of sub-clinical cardiovascular health for this age group. However, the lack of prospective CRP–fatigue associations for participants aged 60 or younger, unless very high CRP measurements are included, suggests that the role of systemic inflammation itself in the development of fatigue, at least in middle-aged, may have been previously overstated.

### Limitations

This analysis has several considerable strengths. Unlike previous longitudinal studies, we excluded participants with CRP >10 mg/L to focus specifically on chronic inflammatory processes. Examining the inflammation–fatigue relationship across the whole adult lifespan for the first time, we were able to describe when an association of fatigue and chronic inflammation becomes apparent. Unlike previous studies, we were able to describe trajectories of CRP–fatigue associations across three follow-up points, considering a wide variety of confounding factors including onset of new disease.

Use of the SF-12's single-item fatigue measure, and not the four-item sub-scale from the SF-36, was a limitation; different relationships could exist between CRP and the other fatigue dimensions of feeling ‘tired’, ‘worn out’, and ‘full of life’. The lack of CRP at follow-up meant the reverse pathway (fatigue to CRP) could not be simultaneously explored, and we could not include diagnoses at baseline of arthritis. The lack of measures such as intima media thickness (for atherosclerosis), objective physical activity, and fatigability in this dataset meant hypotheses around age-related differences in sub-clinical health, activity levels, and reporting could not be further explored. Investigation in other datasets will be required to clarify intervening processes linking CRP and fatigue in older individuals.

## Conclusions

In a large community sample, self-reported fatigue was clearly associated with the inflammatory marker CRP only for older participants (aged 61–98). This may reflect a link in older people via sub-clinical illnesses, which become more common through the lifespan. However, the lack of CRP–fatigue associations at younger ages suggests involvement of systemic inflammation *per se* in fatigue development among working-age adults may be less than previously thought, or emerge only at very high CRP levels. Previously reported associations in mid-life may have been overestimated, inflated by infection-related sickness behaviour or undiagnosed illnesses at baseline. Further research is needed to clarify mechanisms responsible for the CRP–fatigue association in older individuals.

## Supplementary material

For supplementary material accompanying this paper visit http://dx.doi.org/10.1017/S0033291717002872. click here to view supplementary material
