# Supplementary material for: Age modification of the relationship between C-reactive protein and fatigue: findings from Understanding Society (UKHLS)
Source: Psychol Med. 2017 Oct 10;48(8):1341–9. doi: 10.1017/S0033291717002872 (PMC6088542; doi:10.1017/S0033291717002872)
Supplement: Supplementary file 1 [file S0033291717002872sup.zip › S0033291717002872sup002.docx]

| **Table S2: Sensitivity analyses^a^: associations of CRP and New-Onset Fatigue 1, 2 and 3 waves later, participants aged 61-98 (N=2,859)** | | | | | | | | | |
| --- | --- | --- | --- | --- | --- | --- | --- | --- | --- |
| *Adjusted for time, season, processing of sample (N=2,859)* | | | | | | | | | |
|  | 1 wave later | | | 2 waves later | | | 3 waves later | | |
| CRP Category | OR | CI | p | OR | CI | p | OR | CI | p |
| Mid (1.00-2.99mg/L) | 1.19 | 0.79-1.78 | 0.41 | 1.31 | 0.88-1.96 | 0.18 | 1.78 | 0.94-3.37 | 0.08 |
| High (3.00-10.0mg/L) | 1.88 | 1.21-2.92 | 0.005 | 2.26 | 1.46-3.49 | <0.001 | 1.66 | 1.08-2.55 | 0.02 |
| *Adjustment for smoking status (N=2,837)* | | | | | | | | | |
|  | 1 wave later | | | 2 wave later | | | 3 wave later | | |
| CRP Category | OR | CI | p | OR | CI | p | OR | CI | p |
| Mid (1.00-2.99mg/L) | 1.19 | 0.79-1.78 | 0.41 | 1.28 | 0.86-1.91 | 0.22 | 1.75 | 0.92-3.34 | 0.09 |
| High (3.00-10.0mg/L) | 1.81 | 1.16-2.82 | 0.01 | 2.19 | 1.42-3.38 | 0.001 | 1.58 | 1.02-2.43 | 0.04 |
| *Adjusted for Hba1c (N=2,663)* | | | | | | | | | |
|  | 1 wave later | | | 2 wave later | | | 3 wave later | | |
| CRP Category | OR | CI | p | OR | CI | p | OR | CI | p |
| Mid (1.00-2.99mg/L) | 1.32 | 0.87-2.00 | 0.20 | 1.25 | 0.83-1.89 | 0.28 | 1.69 | 0.87-3.26 | 0.12 |
| High (3.00-10.0mg/L) | 1.83 | 1.16-2.91 | 0.01 | 2.12 | 1.35-3.34 | 0.001 | 1.51 | 0.96-2.35 | 0.07 |
| *Adjusted for blood pressure (N=1,810)* | | | | | | | | | |
|  | 1 wave later | | | 2 wave later | | | 3 wave later | | |
| CRP Category | OR | CI | p | OR | CI | p | OR | CI | p |
| Mid (1.00-2.99mg/L) | 1.19 | 0.73-1.96 | 0.49 | 1.58 | 0.95-2.62 | 0.077 | 2.65 | 1.24-5.66 | 0.01 |
| High (3.00-10.0mg/L) | 1.85 | 1.06-3.24 | 0.03 | 3.21 | 1.84-5.61 | <0.001 | 1.75 | 1.02-3.00 | 0.04 |
| *Adjustment for anaemia status (N=2,654)* | | | | | | | | | |
|  | 1 wave later | | | 2 waves later | | | 3 waves later | | |
| CRP Category | OR | CI | p | OR | CI | p | OR | CI | p |
| Mid (1.00-2.99mg/L) | 1.35 | 0.88-2.06 | 0.17 | 1.27 | 0.84-1.92 | 0.25 | 1.66 | 0.85-3.21 | 0.14 |
| High (3.00-10.0mg/L) | 1.93 | 1.21-3.08 | 0.005 | 2.15 | 1.36-3.39 | 0.001 | 1.51 | 0.97-2.37 | 0.07 |
| *Adjustment for drinking frequency (N=2,668)* | | | | | | | | | |
|  | 1 wave later | | | 2 waves later | | | 3 waves later | | |
| CRP Category | OR | CI | p | OR | CI | p | OR | CI | p |
| Mid (1.00-2.99mg/L) | 1.22 | 0.79-1.87 | 0.37 | 1.21 | 0.80-1.82 | 0.37 | 1.63 | 0.84-3.19 | 0.15 |
| High (3.00-10.0mg/L) | 1.65 | 1.02-2.66 | 0.042 | 2.09 | 1.32-3.31 | 0.002 | 1.47 | 0.94-2.31 | 0.09 |
| *Adjustment for drinking heaviness (N=2,580)* | | | | | | | | | |
|  | 1 wave later | | | 2 waves later | | | 3 waves later | | |
| CRP Category | OR | CI | p | OR | CI | p | OR | CI | p |
| Mid (1.00-2.99mg/L) | 1.24 | 0.81-1.91 | 0.32 | 1.30 | 0.85-1.97 | 0.22 | 1.67 | 0.85-3.27 | 0.14 |
| High (3.00-10.0mg/L) | 1.69 | 1.05-2.73 | 0.03 | 2.19 | 1.38-3.48 | 0.001 | 1.55 | 0.98-2.45 | 0.06 |
| *Exclusion of non-white participants (N=2,855)* | | | | | | | | | |
|  | 1 wave later | | | 2 waves later | | | 3 waves later | | |
| CRP Category | OR | CI | p | OR | CI | p | OR | CI | p |
| Mid (1.00-2.99mg/L) | 1.22 | 0.81-1.84 | 0.35 | 1.30 | 0.87-1.94 | 0.21 | 1.83 | 0.94-3.53 | 0.07 |
| High (3.00-10.0mg/L) | 1.98 | 1.27-3.10 | 0.003 | 2.26 | 1.45-3.52 | <0.001 | 1.63 | 1.05-2.52 | 0.03 |
| *Exclusion for anti-inflammatory medications (N=1,747)* | | | | | | | | | |
|  | 1 wave later | | | 2 waves later | | | 3 waves later | | |
| CRP Category | OR | CI | p | OR | CI | p | OR | CI | p |
| Mid (1.00-2.99mg/L) | 1.11 | 0.63-1.95 | 0.72 | 1.33 | 0.75-2.36 | 0.34 | 1.76 | 0.82-3.81 | 0.15 |
| High (3.00-10.0mg/L) | 2.02 | 1.12-3.65 | 0.02 | 3.02 | 1.64-5.55 | <0.001 | 1.75 | 1.00-3.05 | 0.05 |
| *Exclusion for Hormone Replacement Therapy/Oral Contraceptives use (N=2,812)* | | | | | | | | | |
|  | 1 wave later | | | 2 waves later | | | 3 waves later | | |
| CRP Category | OR | CI | p | OR | CI | p | OR | CI | p |
| Mid (1.00-2.99mg/L) | 1.15 | 0.77-1.74 | 0.49 | 1.32 | 0.88-1.98 | 0.18 | 1.93 | 0.99-3.76 | 0.05 |
| High (3.00-10.0mg/L) | 1.80 | 1.15-2.80 | 0.01 | 2.34 | 1.50-3.65 | <0.001 | 1.61 | 1.04-2.49 | 0.03 |
| *Exclusion of current smokers (N=2,544)* | | | | | | | | | |
|  | 1 wave later | | | 2 waves later | | | 3 waves later | | |
| CRP Category | OR | CI | p | OR | CI | p | OR | CI | p |
| Mid (1.00-2.99mg/L) | 1.27 | 0.83-1.96 | 0.28 | 1.29 | 0.84-1.96 | 0.24 | 1.92 | 0.94-3.92 | 0.07 |
| High (3.00-10.0mg/L) | 1.87 | 1.16-3.04 | 0.01 | 1.83 | 1.14-2.95 | 0.01 | 1.56 | 0.99-2.48 | 0.06 |
| ^a^All models adjust for age in years, gender, household income, BMI and longterm illness at baseline, new somatic illness and psychological distress at follow-up. Low CRP (<1.00mg/L) is the reference in all models. | | | | | | | | | |
