# Supplementary material for: Age modification of the relationship between C-reactive protein and fatigue: findings from Understanding Society (UKHLS)
Source: Psychol Med. 2017 Oct 10;48(8):1341–9. doi: 10.1017/S0033291717002872 (PMC6088542; doi:10.1017/S0033291717002872)
Supplement: Supplementary file 1 [file S0033291717002872sup.zip › S0033291717002872sup001.docx]

| **Table S1: Sensitivity analyses^a^: associations of CRP and Fatigue 1, 2 and 3 waves later, participants aged 61-98 (N=3,545)** | | | | | | | | | | |
| --- | --- | --- | --- | --- | --- | --- | --- | --- | --- | --- |
| *Adjusted for time, season, processing of sample (N=3,545)* | | | | | | | | | | |
|  | 1 wave later | | | 2 waves later | | | 3 waves later | | | |
| CRP Category | Coeff | CI | p | Coeff | CI | p | Coeff | CI | p | |
| Mid (1.00-2.99mg/L) | 0.02 | -0.03-0.08 | 0.46 | 0.07 | 0.01-0.13 | 0.03 | 0.09 | 0.00-0.18 | <0.05 | |
| High (3.00-10.0mg/L) | 0.16 | 0.10-0.23 | <0.001 | 0.14 | 0.07-0.21 | <0.001 | 0.08 | 0.01-0.16 | 0.03 | |
| *Adjustment for smoking status (N=3,519)* | | | | | | | | | | |
|  | 1 wave later | | | 2 waves later | | | 3 waves later | | | |
| CRP Category | Coeff | CI | p | Coeff | CI | p | Coeff | CI | p | |
| Mid (1.00-2.99mg/L) | 0.02 | -0.03-0.08 | 0.45 | 0.06 | 0.00-0.13 | 0.04 | 0.09 | 0.00-0.18 | 0.06 | |
| High (3.00-10.0mg/L) | 0.16 | 0.09-0.22 | 0.00 | 0.13 | 0.06-0.21 | 0.00 | 0.08 | 0.00-0.15 | 0.05 | |
| *Adjusted for Hba1c (N=3,287)* | | | | | | | | | | |
|  | 1 wave later | | | 2 waves later | | | 3 waves later | | | |
| CRP Category | Coeff | CI | p | Coeff | CI | p | Coeff | CI | p | |
| Mid (1.00-2.99mg/L) | 0.02 | -0.03-0.08 | 0.42 | 0.06 | 0.00-0.13 | 0.04 | 0.09 | 0.00-0.18 | 0.06 | |
| High (3.00-10.0mg/L) | 0.16 | 0.09-0.23 | <0.001 | 0.13 | 0.06-0.21 | 0.001 | 0.08 | 0.00-0.16 | 0.04 | |
| *Adjusted for blood pressure (N=2,205)* | | | | | | | | | | |
|  | 1 wave later | | | 2 waves later | | | 3 waves later | | | |
| CRP Category | Coeff | CI | p | Coeff | CI | p | Coeff | CI | p | |
| Mid (1.00-2.99mg/L) | 0.04 | -0.03-0.11 | 0.24 | 0.09 | 0.02-0.16 | 0.02 | 0.13 | 0.03-0.24 | 0.01 | |
| High (3.00-10.0mg/L) | 0.17 | 0.08-0.26 | <0.001 | 0.15 | 0.06-0.25 | 0.001 | 0.08 | -0.01-0.18 | 0.09 | |
| *Adjustment for anaemia status (N=3,278)* | | | | | | | | | | |
|  | 1 wave later | | | 2 waves later | | | 3 waves later | | | |
| CRP Category | Coeff | CI | p | Coeff | CI | p | Coeff | CI | p | |
| Mid (1.00-2.99mg/L) | 0.03 | -0.03-0.08 | 0.39 | 0.07 | 0.01-0.13 | 0.03 | 0.09 | 0.00-0.18 | | 0.06 |
| High (3.00-10.0mg/L) | 0.17 | 0.10-0.24 | <0.001 | 0.14 | 0.06-0.22 | <0.001 | 0.09 | 0.01-0.17 | | 0.03 |
| *Adjustment for drinking frequency (N=3,272)* | | | | | | | | | | |
|  | 1 wave later | | | 2 waves later | | | 3 waves later | | | |
| CRP Category | Coeff | CI | p | Coeff | CI | p | Coeff | CI | p | |
| Mid (1.00-2.99mg/L) | 0.03 | -0.02-0.09 | 0.25 | 0.07 | 0.01-0.13 | 0.03 | 0.09 | 0.00-0.19 | 0.05 | |
| High (3.00-10.0mg/L) | 0.13 | 0.06-0.20 | <0.001 | 0.14 | 0.07-0.22 | <0.001 | 0.09 | 0.01-0.16 | 0.03 | |
| *Adjustment for drinking heaviness (N=3,170)* | | | | | | | | | | |
|  | 1 wave later | | | 2 waves later | | | 3 waves later | | | |
| CRP Category | Coeff | CI | p | Coeff | CI | p | Coeff | CI | p | |
| Mid (1.00-2.99mg/L) | 0.04 | -0.02-0.10 | 0.21 | 0.08 | 0.02-0.14 | 0.01 | 0.10 | 0.01-0.19 | 0.04 | |
| High (3.00-10.0mg/L) | 0.13 | 0.06-0.20 | <0.001 | 0.15 | 0.07-0.22 | <0.001 | 0.09 | 0.02-0.17 | 0.02 | |
| *Exclusion of non-white participants (N=3,497)* | | | | | | | | | | |
|  | 1 wave later | | | 2 waves later | | | 3 waves later | | | |
| CRP Category | Coeff | CI | p | Coeff | CI | p | Coeff | CI | p | |
| Mid (1.00-2.99mg/L) | 0.02 | -0.03-0.08 | 0.40 | 0.07 | 0.01-0.13 | 0.03 | 0.09 | 0.00-0.18 | 0.04 | |
| High (3.00-10.0mg/L) | 0.17 | 0.10-0.24 | <0.001 | 0.14 | 0.06-0.21 | <0.001 | 0.08 | 0.01-0.16 | 0.04 | |
| *Exclusion for anti-inflammatory medications (N=2,049)* | | | | | | | | | | |
|  | 1 wave later | | | 2 waves later | | | 3 waves later | | | |
| CRP Category | Coeff | CI | p | Coeff | CI | p | Coeff | CI | p | |
| Mid (1.00-2.99mg/L) | 0.03 | -0.04-0.10 | 0.44 | 0.10 | 0.03-0.18 | 0.009 | 0.13 | 0.03-0.23 | 0.01 | |
| High (3.00-10.0mg/L) | 0.15 | 0.06-0.24 | 0.001 | 0.16 | 0.07-0.26 | 0.001 | 0.12 | 0.02-0.22 | 0.02 | |
| *Exclusion for Hormone Replacement Therapy/Oral Contraceptives use (N=3,491)* | | | | | | | | | | |
|  | 1 wave later | | | 2 waves later | | | 3 waves later | | | |
| CRP Category | Coeff | CI | p | Coeff | CI | p | Coeff | CI | p | |
| Mid (1.00-2.99mg/L) | 0.02 | -0.04-0.08 | 0.51 | 0.07 | 0.01-0.13 | 0.02 | 0.11 | 0.01-0.20 | 0.02 | |
| High (3.00-10.0mg/L) | 0.16 | 0.09-0.23 | <0.001 | 0.14 | 0.07-0.22 | <0.001 | 0.08 | 0.00-0.15 | 0.04 | |
| *Exclusion of current smokers (N=3,135)* | | | | | | | | | | |
|  | 1 wave later | | | 2 waves later | | | 3 waves later | | | |
| CRP Category | Coeff | CI | p | Coeff | CI | p | Coeff | CI | p | |
| Mid (1.00-2.99mg/L) | 0.02 | -0.04-0.08 | 0.49 | 0.07 | 0.00-0.13 | 0.04 | 0.09 | 0.00-0.19 | 0.06 | |
| High (3.00-10.0mg/L) | 0.13 | 0.05-0.20 | 0.001 | 0.10 | 0.02-0.18 | 0.01 | 0.06 | -0.02-0.14 | 0.14 | |
| ^a^All models adjust for age in years, gender, household income, BMI and longterm illness at baseline, new somatic illness and psychological distress at follow-up. Low CRP (<1.00mg/L) is the reference in all models | | | | | | | | | | |
